# Hearing loss secondary to novel variants of the *KCNQ4* gene

**DOI:** 10.1007/s00405-025-09288-x

**Published:** 2025-04-03

**Authors:** Rocío González-Aguado, Julia Fernández-Enseñat, Esther Onecha, Carmelo Morales-Angulo

**Affiliations:** 1 Department of Otolaryngology, Hospital Universitario Marqués de Valdecilla, Santander, Cantabria Spain; 2 Department of Genetics, Hospital Universitario Marqués de Valdecilla, Santander, Cantabria Spain; 3 Department of Otolaryngology, Hospital Universitario Marqués de Valdecilla, Santander, Cantabria Spain; 4 Faculty of Medicine, University of Cantabria, Cantabria, Spain; 5 Cell Cycle, Stem Cell Fate and Cancer Laboratory, Institute for Research Marqués de Valdecilla (IDIVAL), 39011 Santander, Spain

**Keywords:** *KCNQ4*, Autosomal dominant, Genetic counselling, Genetic hearing loss, Variants, Sensorineural hearing loss

## Abstract

**Purpose:**

Heterozygous variants of the *KCNQ4* gene are associated with isolated sensorineural hearing loss (DFNA2A). This study aimed to determine the frequency and clinical characteristics of pathogenic, likely pathogenic, and uncertain variants in the *KCNQ4* gene among patients with sensorineural hearing loss of unknown origin in North Spain.

**Methods:**

We conducted a prospective observational study of patients with sensorineural hearing loss of unknown etiology at a tertiary hospital over six years. Next-generation sequencing carried out with a panel of genes was used to identify genetic variants related to both syndromic and non-syndromic hearing loss.

**Results:**

Among 370 patients, seven (1.89%) harbored pathogenic or likely pathogenic variants in the *KCNQ4* gene: c.777_778delinsCC, c.626 T > G, and c.778G > C. None of these variants had been previously described. One patient also had a variant of uncertain significance (c.419 T > C). All patients exhibited progressive bilateral sensorineural hearing loss, predominantly at high frequencies, with variable onset and severity. None reported dizziness or vertigo. Five patients used hearing aids, and one received a cochlear implant with good results.

**Conclusions:**

*KCNQ4* gene variants are rare in Cantabria, present in less than 2% of patients with sensorineural hearing loss of unknown origin. Although most variants identified in our study had not been previously described, the observed phenotype aligned with the typical presentation: bilateral, progressive sensorineural hearing loss with variable onset and severity. Some patients may benefit from cochlear implants.

## Introduction

Potassium voltage-gated channel (*KCNQ*) genes encode proteins that are part of potassium channels and act as cotransporters, playing an important role in the regulation of neuronal excitability, particularly in the sensory cells of the cochlea [1]. Pathogenic variants of these genes lead to dysfunctional K + regulation in the inner ear, resulting in ineffective transmission of nerve electrical signals [1] and, ultimately, syndromic or non-syndromic hearing loss [2, 3].

Variants of the *KCNQ1* gene, which are transmitted in an autosomal recessive (AR) manner, can cause Jervell and Lange-Nielsen syndrome, characterized by sensorineural hearing loss and an increased QT interval [4]. Conversely, heterozygous variants of the *KCNQ4* gene, transmitted in an autosomal dominant (AD) manner, can cause isolated sensorineural hearing loss (DFNA2A) [5–7]. Non-syndromic hearing loss secondary to heterozygous variants in the *KCNQ4* gene has been described in populations from all continents [7–11]. Although the phenotypes of several described families are similar [7], there is considerable heterogeneity related to the different genetic mutations identified so far [12].

The objective of our study was to determine the prevalence of *KCNQ4* gene variants in patients with sensorineural hearing loss of unknown etiology in the Community of Cantabria and to understand the clinical characteristics of patients carrying these variants, with the aim of assisting in genetic counseling by correlating genotype and phenotype.

Methods.

An observational, prospective, and descriptive study was conducted from 2018 to 2024 at the Otorhinolaryngology Department of a tertiary hospital of North Spain. The study focused on patients with bilateral sensorineural hearing loss of unknown etiology.

The following criteria (all of them should meet) were used for patient selection in the genetic study:

- Bilateral sensorineural hearing loss.
- Suspected genetic origin of the hearing loss based on the presence of a family history of hearing loss, characteristic hearing profile (bilateral U-shaped or ascending audiogram), or bilateral sensorineural hearing loss more severe than expected for age-related hearing loss (presbycusis).
- Unknown etiology.

For each individual, a form was completed that recorded key information from the clinical history and family history of hearing loss.

Regarding hearing loss, factors such as the age of onset, mode of onset (sudden, fluctuant or progressive), laterality (unilateral or bilateral, symmetric or asymmetric), evolution (stable or progressive), and the presence of other symptoms were considered. Otological symptoms associated with hearing loss, including tinnitus, vertigo, otalgia, or otorrhea, were also recorded.

A systemic examination was performed to rule out abnormalities associated with hearing loss. In cases reporting dizziness or vertigo, an examination of the vestibular system was carried out.

The degree of hearing loss on tonal audiometry was classified according to the criteria established by the American Speech-Language-Hearing Association [13]: slight (16–25 decibels [dB]), mild (26–40 dB), moderate (41–55 dB), moderately severe (56–70 dB), severe (71–90 dB), or profound (91 dB or greater). The configuration of hearing loss, as determined by audiometric analysis, was categorized as sloping, flat, rising (low frequency), or midfrequency (cookie-bite) loss [14].

In patients with variants in the *KCNQ4* gene, additional tests were requested, including a complete blood count, biochemical profile, elemental profile, urinary sediment analysis, and electrocardiogram, to rule out syndromic hearing loss. In selected cases, the clinical study was further completed by performing auditory brainstem evoked responses (ABR), vestibular test, computed tomography (CT) or magnetic resonance imaging (MRI) of the petrous bone..

Genetic analysis was conducted through next-generation sequencing (NGS) carried out using a panel of genes associated with hearing loss including the *KCNQ4* gene. The sequencing workflow was performed by capturing genes of interest using RNA probes with SureSelect technology (Agilent Technologies, Inc. San Diego, California, USA) and subsequent sequencing using the Miseq sequencer from Illumina (San Diego, California, USA.).

The bioinformatics analysis was performed with Alissa software that integrates the programs BWAmem for alignment of sequences against the reference genome (assembly hg19) and SAMtools was used to generate BAM files. Filtering of genetic variants was carried out according to with alignment and genotyping quality metrics. The detection of copy number variations was performed with an adapted version of the software DECoN (PMID:28,459,104).

Those variants identified from alignments with low mapping quality, variants with strand bias, thus such as the variants published as benign and probably benign by multiple subscribers in the database ClinVar and HGMD were discarded. Variants were annotated using several databases containing functional (Ensembl, CCDS, RefSeq, Pfam), populational (dbSNP, gnomAD, 1000 Genomes, ESP6500, ExAC), and disease-related (Clinvar, HGMD professional) information. And databases of hereditary diseases OMIM, Orphanet and GeneReviews.

The classification of the variants was carried out in 5 categories: pathogenic (P), likely pathogenic (LP), meaning uncertain (VUS), likely benign (LB) and benign. Following the recommendations of the ACMG, and expert specification of the ACMG/AMP variant interpretation guidelines for genetic hearing loss. (PMID:30,311,386).

### Statistical analysis

A descriptive statistical analysis was performed on the studied variables. To quantify hearing loss progression, a linear regression model was created, relating age (independent variable) to the degree of hearing loss (dependent variable) using Microsoft Excel.

The study was done in accordance with the Code of Ethics of the World Medical Association (Helsinki Declaration). The study was approved by the Ethics Committee for Research with Medicines and Health Products of Cantabria (CEIM), internal code: 2024.104 (Acta 8/2024 de 22/03/2024).. The investigators have obtained written informed consent from each participant.

## Results

Of the 370 patients studied, a *KCNQ4* gene variant was detected in 8 patients (2.16% of the total; 1.8% for pathogenic/likely pathogenic variants), aged 37 to 77 years, from 8 apparently unrelated families.

**Fig. 1:**
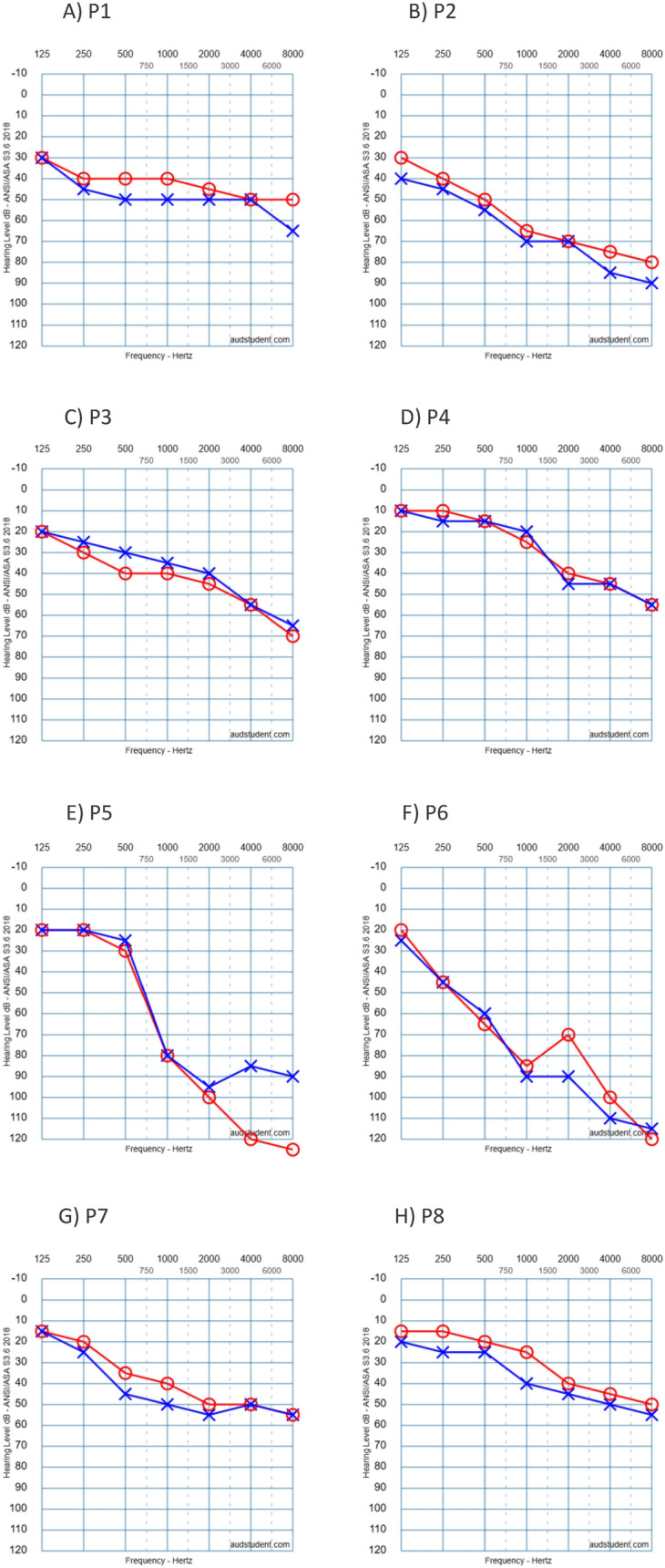
First audiometry of all patients with a variant in the KCNQ4 gene

**Fig. 2:**
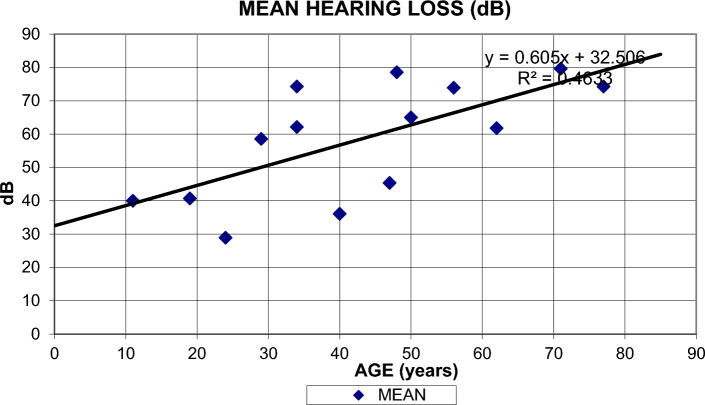
It depicts hearing loss degree (mean thresholds) versus age, with a black linear regression line

The pathogenic/likely pathogenic (P/LP) variants found were c.777_778delinsCC, c.626 T > G, and c.778G > C. Additionally, one variant of uncertain significance (VUS) was identified: c.419 T > C. All variants displayed an autosomal dominant (AD) inheritance pattern. The clinical and genetic data are summarized in Table 1. All patients presented with mild to profound, progressive, bilateral sensorineural hearing loss across all frequencies, with a predominance of high frequencies (Fig. 1). Comparing recent audiograms with earlier ones, we represented the degree of hearing loss for all individuals carrying *KCNQ4* gene variants as the mean threshold across all frequencies, plotted against age (Fig. 2). Despite the data variability, a simple linear regression model was applied to predict the degree of hearing loss based on age, minimizing the differences between observed and expected values:

**Table 1:** Demographic, clinical and genetic characteristics of patients with a variant in the KCNQ4 gene

Patient Nº/ Age/sex	FB/ inheritance pattern	Age of onset	Progression	Audiogram configuration	Degree of HL	Vestibular symptoms/VT	CT/ MRI	Genetic Variant/protein (classification)	Treatment
P1/ 37/Female	Yes/AD	Unknown	Yes	Sloping	OD: severe OI: moderately severe	No, V-HIT: normal	Normal	c.777_778delinsCC, p.Glu260Gln (pathogenic)	HA Unilateral CI
P2/ 41/Female	Yes/AD	10–20	Yes	Sloping	Moderately severe	No	No	c.777_778delinsCC, p.Glu260Gln (pathogenic)	No
P3/ 77/Female	Yes/AD	30	Yes	Moderate Sloping	Severe	No, V-HIT: normal	Normal	c.626 T > G, p.leu209Arg (pathogenic)	HA. He rejects a CI
P4/ 57/Female	Yes/AD	30	Yes	Sloping	Moderately severe/profound	No	No	c.778G > C, p.Glu260Gln (pathogenic)	HA. He rejects a CI
P5/ 71/Male	Yes/AD	30–40	Yes	Sloping	Moderate/severe	No	Normal	c.626 T > G, p.leu209Arg (pathogenic)	HA He rejects a CI (his brother had a CI with postoperative vertigo)
P6/ 49/Male	Yes/AD	20–30	Yes	Sloping	Severe/profound	No	No	c.778G > C, p.Glu260Gln (pathogenic)	HA (candidate to CI)
P7/ 38/Male	Yes/AD	10–20	Yes	Sloping	Moderate /severe	No	No	c.778G > C, p.Glu260Gln (pathogenic)	He didn´t tolerate HA due to increased tinnitus
P8/ 43/Male	Yes/AD	10–15	Yes	Sloping	Mild/ Moderate	No	No	c.419 T > C, p.lle140Thr (VUS)	No

*FB* family background

*VT* vestibular test

*HL* hearing loss

CI cochlear implant

*VUS* variant of unknown significance

*AD* autosomal dominant

Degree of hearing loss (MEAN) in dB = 0.605 × AGE (years) + 32.506 (R^2^ = 0.4633).

The onset of hearing loss was variable, predominantly occurring in adulthood, and in 2 cases, it was slightly asymmetrical.

Auditory brainstem response (ABR) testing performed on one of the patients demonstrated a pattern compatible with cochlear sensorineural hearing loss. No patient reported symptoms suggestive of vestibular pathology. Videonystagmography and video head impulse tests performed in two patients were normal.

Computed tomography was conducted in 3 patients, revealing no evidence of inner ear malformations. In none of the cases were findings suggestive of a syndromic form observed. The treatment received included hearing aid adaptation for 6 of the patients with pathogenic variants, but one of them subsequently required a cochlear implant (CI), with a good response in terms of speech understanding. Additionally, 4 patients were candidates for CI placement but declined the surgical intervention; one of them cited a concern due to a brother who, after CI placement, experienced recurrent episodes of vertigo.

## Discussion

The frequency of P/LP variants of the *KCNQ4* gene in our community was 1.8% among cases with bilateral sensorineural hearing loss of unknown origin, which is somewhat similar to rates found in other Spanish regions [1, 15] and in other European studies [16].

On the other hand, Naito et al. identified pathogenic variants in the *KCNQ4* gene in 19 families (16.61%) among a study involving 287 Japanese probands with AD non-syndromic hearing loss, detecting 7 different variants. Among these families, the c.211delC variant was found in 13 out of 19 families, with patients carrying it exhibiting progressive high-frequency hearing loss starting between 3 and 40 years of age. Haplotype analysis suggested a common ancestor for these cases [17].

In a large genetic study conducted in Taiwan on hereditary hearing loss, researchers initially ruled out variants of the connexin 26 gene and the 1555A > G variant of the *MTRNR1* gene, subsequently finding 5 out of 86 cases with pathogenic variants of the *KCNQ4* gene [18]. Therefore, pathogenic variants in the *KCNQ4* gene are not uncommon in some populations.

To date, more than 70 genetic variants with diverse clinical phenotypes have been identified in the KCNQ4 gene [19]. Except the VUS variant (c.419 T > C) all others found in our study were novel. The variants c.777_778delinsCC and c.778G > C both result in the same effect at the protein level, causing a change from glutamic acid to glutamine at position 260 of the protein. Additionally, the variant c.626 T > G leads to an amino acid change from leucine to arginine at position 209. The complete protein comprises 695 amino acids. Furthermore, the variant of uncertain significance (VUS) c.419 T > C results in an amino acid change from isoleucine to threonine at a specific protein position.

All individuals in our series had bilateral sensorineural hearing loss predominantly affecting high frequencies, with variable intensity and onset, typically occurring between the 2nd and 3rd decades of life. This hearing loss progresses slowly to moderate-to-severe (and even profound in high frequencies), subsequently affecting mid and low frequencies. Homma et al. found that missense variants are associated with earlier onset and pan-tonal hearing loss, while deletions lead to later onset hearing loss, predominantly in high frequencies [19]. Cases with onset in the first decade of life have been described, although most typically begin in the second and third decades, as seen in our study [11, 20, 21].

Hearing loss progression in our cohort was universally clear but slow. Other authors have found that progression of hearing loss is also variable within families and depends on the specific variant, with some variants (e.g., p.G285S) demonstrating significantly accelerated progression of hearing loss compared to others [21]. This variability is important to consider in genetic counseling and prognosis. Different pathogenic mechanisms are proposed to explain phenotypic differences, including haploinsufficiency in deletion mutations and dominant negative effects in nonsense mutations [21].

Neurophysiological studies suggest that cochlear damage is involved. None of our patients presented with balance disorders, and vestibular complementary tests performed on two patients showed no involvement of the vestibular system. Furthermore, most previous studies have found no vestibular alterations associated with pathogenic variants in *KCNQ4*, suggesting they do not lead to vestibular dysfunction [11]. However, Yen et al. reported that 2 out of 12 patients (16%) with the c.546C > G pathogenic variant in the *KCNQ4* gene experienced vertigo [22]. Clinical findings accompanying hearing loss were also not observed in our study or in previous publications, indicating non-syndromic forms.

In our series, only three patients underwent imaging tests of the inner ear, which did not show radiological abnormalities, similar to findings previously described in mutations of the *KCNQ4* gene that do not demonstrate temporal bone malformations [7]. Most of the patients initially received hearing aids, but five of them were candidates for CIs), and only one accepted it. Following the procedure, there was a notable improvement in speech comprehension. There are few references to CIs in patients with *KCNQ4* gene mutations. Selignman et al. conducted a large series of genetic studies involving 459 cochlear implanted patients, among whom 2 were found to have pathogenic variants in the *KCNQ4* gene. Interestingly, the study did not provide commentary on the auditory outcomes in these cases [23].

Nam et al. discovered that valproic acid inhibited the progression of sensorineural hearing loss in a *KCNQ4* p.W276S variant model, suggesting it could be a candidate drug to delay hearing loss in some patients [24]. Meanwhile, recent advances have enabled the successful use of gene therapy for variants in genes such as otoferlin [25]. Additionally, preliminary results in mice indicate the possibility of restoring KCNQ4 expression and function in outer hair cells through targeted therapies, specifically CRISPR-based gene therapy, for DFNA2. This approach holds promise for preventing and treating age-related hearing loss caused by KCNQ4 variants [26].

The limitations of our study include the small number of patients and the lack of complementary studies on affected family members, which would have reinforced the clinical and genetic findings. Additionally, since the study was conducted in a region of northern Spain, the results may not be generalizable to other parts of the country or beyond. Furthermore, a control group was not used to assess the prevalence of the variants in the general population, nor was a functional analysis conducted to evaluate the impact of the identified KCNQ4 variants. However, we believe our study is of interest, as there are few published studies (none in our country) on the auditory phenotype of patients with mutations in the *KCNQ4* gene, and we described variants that have not been previously published.

## Conclusions

Variants in the *KCNQ4* gene within our community are infrequent. We have detected new variants that result in a similar phenotype to previous pathogenic variants: bilateral post-lingual sensorineural hearing loss with AD transmission and no vestibular impairment. Some patients with *KCNQ4* variants are good candidates for cochlear implantation.
